# Clinical, Diagnostic, and Therapeutic Aspects of Growth Hormone Deficiency During the Transition Period: Review of the Literature

**DOI:** 10.3389/fendo.2021.634288

**Published:** 2021-02-24

**Authors:** Matteo Spaziani, Chiara Tarantino, Natascia Tahani, Daniele Gianfrilli, Emilia Sbardella, Andrea M. Isidori, Andrea Lenzi, Antonio F. Radicioni

**Affiliations:** ^1^ Section of Medical Pathophysiology and Endocrinology, Department of Experimental Medicine, Sapienza University of Rome, Rome, Italy; ^2^ Centre for Rare Diseases, Policlinico Umberto I, Rome, Italy; ^3^ Department of Diabetes, Endocrinology and Metabolism, Queen Elizabeth Hospital, University Hospitals Birmingham NHS Foundation Trust, Birmingham, United Kingdom

**Keywords:** transition period, bone, body composition, metabolism, gonad function, growth hormone deficiency, cancer survivors

## Abstract

The role of growth hormone (GH) during childhood and adulthood is well established. Once final stature is reached, GH continues to act during the transition, the period between adolescence and adulthood in which most somatic and psychological development is obtained. The achievement of peak bone mass represents the most relevant aspect of GH action during the transition period; however, equally clear is its influence on body composition and metabolic profile and, probably, in the achievement of a complete gonadal and sexual maturation. Despite this, there are still some aspects that often make clinical practice difficult and uncertain, in particular in evaluating a possible persistence of GH deficiency once final stature has been reached. It is also essential to identify which subjects should undergo re-testing and, possibly, replacement therapy, and the definition of unambiguous criteria for therapeutic success. Moreover, even during the transition phase, the relationship between GH substitution therapy and cancer survival is of considerable interest. In view of the above, the aim of this paper is to clarify these relevant issues through a detailed analysis of the literature, with particular attention to the clinical, diagnostic and therapeutic aspects.

## Introduction

The importance and effectiveness of somatotropic hormone replacement therapy (rh-GH) in children with low stature and/or linear growth decline associated with growth hormone deficiency (GHD) is well established. In childhood, there is a diagnostic procedural standard and therapeutic success can be easily evaluated based on the increase in the linear growth rate and the repositioning in the family height target. Nevertheless, there are not the same certainties during the transition period, both in diagnostic and therapeutic terms, even though it has been widely debated in the last 2 decades (1).

The transition phase begins with the achievement of final height and ends with the achievement of peak bone mass. The mean age of the transition onset differs according to sex and the achievement of Prader’s pubertal stage 5 or final adult height. According to Prader, in pubertal stage 5, the mean age is 14.7 ± 2.2 years in boys and 14.0 ± 2.4 years in girls; when considering the final height, the mean age is 16.8 ± 2.2 years in boys and 15.2 ± 2.0 years in girls, and in both cases with a growth velocity <1.5–2 cm/year (2). The end of the transition period usually corresponds with peak bone mass, which occurs at an average age of 23.1 years in males and 19.9 years in females (3). Other Authors define the end of the transition by referring to the sleep chronotype, A percentage of between 25 and 100% of subjects, which occurs at a mean age of 20.9 years for males or 19.5 years for females (4).

The aim of this review is to address the main questions and doubts related to transition, focusing on the diagnostic, clinical and therapeutic tools.

## GHD Diagnosis in Transition: The Importance of Re-Testing

A percentage of between 25% and 100% of subjects with previous childhood-onset GHD (CO-GHD) display a normalization of GH secretion when undergoing re-testing (5).

It is usually recommended 1–3 months between the end of the childhood rh-GH and the time of re-testing (6). This period should not exceed 6 months (5, 7). Of note, not all subjects need to be re-tested: patients with more than two pituitary hormone deficits; isolated GHD associated with an identified mutation and or a specific pituitary/hypothalamic structural defect, except for an ectopic posterior pituitary; the presence of a transcription-factor mutation (6, 8). The positive predictive value for GHD in adulthood is 96% in the presence of three or more pituitary hormone deficiencies and even 99% in the case of four hormone deficits (9). Re-testing should be carried out in the following cases: idiopathic isolated GHD with or without a small pituitary/ectopic posterior pituitary; GHD associated with only one other pituitary hormone deficiency; patients previously treated with radiation therapy.

**Table 1** provides a systematic list of the different available provocative tests, with their respective cut-off values. The gold standard is the Insulin Tolerance Test (ITT) (10–12). In the case of contraindications, such as seizures or confirmed cerebrovascular and cardiovascular risk factors, an alternative is the Glucagon test (10, 14). Another tool used for the diagnosis of GHD is the Arginine test, but it has been shown not to be very effective (11). Finally, the GHRH+Arginine test is not recommended in case of idiopathic GHD, because it may result in a false-positive response (8, 10). In addition, a 2005 study showed that this test should not be used for the diagnosis of GHD in those adults treated with cranial irradiation (CRT) for childhood acute lymphoblastic leukemia, as it could result in a false-positive response (15). For this test, the Italian Medical Agency set the cut-off value to 19 μg/L (16). A recent study investigated the diagnostic accuracy of the GHRH+Arginine test concluding that it should provide specific cut-off points depending on the pathological condition, such as congenital GHD, isolated GHD, multiple pituitary hormone defects and cancer survivors (17).

**Table 1:** List of the different available GH provocative tests, whit their respective cut-off values.

Test	BMI	Cut-off	References
GHRH + Arginine test	BMI <25 kg/m2	<11 μg/L	(8, 10)
	BMI 25–30 kg/m2	<8 μg/L	(8, 10)
	BMI >30 kg/m2	<4 μg/L	(8, 10)
		<19 μg/L	(11)
Insulin Tolerance Test (ITT)		<5 μg/L	(10)
		<6,1 μg/L	(12)
		<5,6 μg/L	(13)
Glucagon test	BMI <25 kg	<3 μg/L	(10, 14)
	BMI 25–30 kg/m2 (high pretest probability)	<3 μg/L	(10, 14)
	BMI 25–30 kg/m2 (low pretest probability)	<1 μg/L	(10, 14)
	BMI >30 kg/m2	<1 μg/L	(10, 14)
Arginine test		<0.4 μg/L	(11)

The possibility of anticipating the time of re-testing, to avoid a hypothetical over-treatment, has been recently investigated by Penta and colleagues (18). The Authors claimed that it would be useful to re-test patients with idiopathic and isolated GHD before reaching final stature or Tanner stage 5, in order to avoid over-treatment.

A special mention should be made of IGF-1: a normal or high IGF1 value alone cannot rule out the diagnosis of GHD (19), as well as a low value alone is not sufficient to make the diagnosis (17, 20). Moreover, to date the chemiluminescence immunoassay should be the preferred method as it is considered the most appropriate and accurate (21).

## The Role of GH During Transition: Effects on Bone, Body Composition, Metabolism, and Gonadal Function

There are numerous studies on the effect and importance of GH during the transition phase. Most of them focus on the influence on bone, metabolism and body composition. In this section, we will also mention the impact of GH on fertility.

### Bone

GH and its mediator IGF-1 have a cumulative effect of raising bone turnover and mass, leading to an increase in endochondral ossification and linear growth.

Although most studies in the literature agree on the role of GH in improving bone quality, there is no unequivocal opinion in this regard. Starting with Randomized Controlled Studies (RCTs), Carroll and Drake respectively showed an increase in total body BMC (TB-BMC) of about 6% and lumbar spine BMC (LS-BMC) of about 5% in treated subjects compared to untreated (22, 23). In a 2-year RCT, an increase of 5% in the total body-bone mineral density (TB-BMD) was found in subjects treated with rh-GH, compared to 1.3% of untreated patients (24). In another 2-year RCT, subjects were randomized either to an adult dose of rh-GH or to a paediatric dose or to a placebo, with an increase of TB-BMD, respectively of 3.3%, 5%, and 1.3% (25). By contrast, another multicentre RCT study, in which 18 GHD subjects were randomized to rh-GH and 15 to placebo, no substantial increase in BMD between the two groups was demonstrated (26). The same findings were obtained by Boot and Hyldstrup (27, 28).

A 2014 cross-sectional study compared 18 subjects with previous CO-GHD diagnosis, who were further subdivided into two sub-groups of nine patients, each based on the persistence or not of GHD after re-testing, with 18 healthy subjects. They showed a reduction in TB-BMD and LS-BMD both in GHD and in non-GHD subjects compared to controls, demonstrating that the previous administration of rh-GH was not sufficient to determine a normal BMD (29).

Of note, CO-GHD patients tend to have smaller bones than control subjects; this may lead to underestimation of Areal BMD assessed by dual energy X-ray absorptiometry (DXA) scan (30, 31) and contribute to discrepancies in the interpretation of results among different investigations. Therefore, it should be considered that those patients with CO-GHD, not treated with rh-GH during childhood, have a significant reduction of the Areal BMD, but a normal Volumetric BMD.

Some studies also assessed the impact of GH on the micro-architectural characteristics of bone. A significant increase in both BMD and in cortical thickness in subjects undergoing rh-GH has been found by Hyldstrup et al (28)., whereas another study found a significant reduction in cortical bone area and thickness in untreated CO-GHD adults compared with AO-GHD (32). Of note, there are no studies that unequivocally demonstrate an increased risk of fractures in GHD subjects during the transition (33, 34).

It should be noted that changes related to eating habits, physical activity and socioeconomic status of patients might occur during rh-GH and they can influence the outcome of bone, metabolic, cardiac and body composition parameters. This makes it more difficult to understand the weight of rh-GH alone in all these changes. Moreover, other pituitary deficiencies and their treatments could play a role in bone metabolism, as the prevalence of fractures is markedly increased in patients with multiple pituitary deficiencies (35).

### Body Composition

Some studies highlighted that a long period off rh-GH induces a consistent increase in the percentage of fat mass (FM) and trunk fat, with a decrease in lean mass (LM) (36–38). During the transition phase, several papers argued that in patients with a GHD persistence and that do not resume rh-GH, there was a reduction in LM and an increase in FM compared to control subjects or those who resumed rh-GH. The LM decreased by about 8%, compared to an average increase of about 15% in FM (22, 39, 40). The resumption of rh-GH, showed a clear improvement, with an increase in LM of about 14% and a decrease in FM of about 7% after 2 years of therapy (24, 41). In contrast, Mauras et al. found no improvement in body composition after 2 years of treatment (26).

Another paper investigated early body composition changes after the rh-GH break in a population with persistent GHD, finding a significant increase in FM especially in those with multiple pituitary deficits, and a significant reduction in cross-sectional muscle area Z-score (42).

GH exerts considerable effects on skeletal muscle, inducing a global effect of muscle growth through the stimulation of free-fatty acids and amino-acid uptake, and the increase of protein synthesis. Rh-GH should not be used during the transition with the only aim of muscle growth, even though several studies have shown clear lower muscle strength in confirmed GHD subjects compared to sufficient or healthy controls (43). A recent study assessed how 12 weeks of resistance exercise alone could improve muscle strength during the transition (44). Interesting is the interconnection between GH, leptin and ghrelin. Leptin, that reduces the sense of appetite, is primarily secreted by white adipose tissue, and its concentration correlates positively with total FM. Ghrelin has an opposite effect and is mainly secreted by P/D1 cells lining the fundus of the stomach and epsilon cells in the pancreas. Ghrelin represents a powerful stimulator to GH secretion, as it is a natural ligand for the GH-secretagogue receptor (45). In a study by Roemmler and colleagues, it was shown that rh-GH was able to reduce the total FM and consequently the leptin levels, albeit there was a no significant increase of ghrelin (46). This connection between GH, ghrelin and leptin paves the way for the possible role of GH in appetite regulation (47).

### Metabolic Aspects and Cardiovascular Risk

Many studies report a worsening of the lipid pattern after discontinuation of the rh-GH (41, 42, 48), although others do not show any substantial difference between patients who resume therapy and subjects who do not (22, 24, 26).

As a general statement we can say that the later treatment begins in childhood and the longer the period off rh-GH lasts during the transition, the worse the lipid profile becomes, with higher total cholesterol and triglyceride levels (49), suggesting that discontinuation of rh-GH during the transition is associated with a pro-atherogenic lipid profile.

Some studies also demonstrated the influence of GH on post-prandial lipid values. Lanes and co-Authors, in 2004, showed an increase in post-prandial triglyceride values (4 h after a high-fat meal) and peripheral inflammatory and fibrinolytic markers in untreated compared to treated subjects (50). In addition, higher levels of basal triglycerides and overlapping values of post-prandial triglycerides compared to an age-matched control group have been found in a population of GHD subjects. This metabolic situation remained unchanged after 4 months of rh-GH (51).

Regarding the effects on glucose metabolism, there are no unequivocal results. While some papers reported an improvement in insulin sensitivity following therapy discontinuation after reaching final stature (52), other studies came to opposite conclusions. One study, in particular, reported a significant increase in insulin resistance following a period of 6 months off rh-GH, which tended to disappear 6 months after the resumption of therapy (53). Some papers highlighted a possible gender-specific difference in carbohydrate metabolism and body composition. Specifically, in a 2004 metanalysis, it is reported that males are more sensitive to the effect of rh-GH on insulin sensitivity (54). Regarding body composition, women seem to be less sensitive than men, and require higher rh-GH doses to achieve the same benefits (55).

There is currently insufficient data to determine whether rh-GH may induce an increased risk of type 2 diabetes mellitus in the future.

The effects of rh-GH on the heart were evaluated in an echocardiographic study, which compared 21 previously treated patients with 21 age- and sex-matched healthy controls. The Authors found that in the 21 studied patients both the heart height and size were lower than in the controls, despite long-term rh-GH during childhood. Then, eight of the 21 patients were subjected again to rh-GH for 15 months during the transition period and they showed a significant increase in left ventricular mass and an improvement in endothelial function within the first 6 months of restarting rh-GH (56). Finally, a 2003 study reported an increase of intima-media thickness (IMT) in subjects with previous CO-GHD, compared to both adult-onset GHD patients and to controls (57); however, this finding was not confirmed in two further studies (53, 58).. An interesting study, carried out in 20 GH-naive Brazilian adults, due to a homozygous mutation in GHRH receptor gene, demonstrated a significant improvement in their lipid metabolic profile after 6 months of rh-GH, while a progressive increase in the number of atherosclerotic carotid plaques was still noted. Moreover, a relevant increase in both cardiac structural parameters (left ventricular mass index, posterior wall, and septum thickness) and carotid IMT was found after 6–12 months of rh-GH suspension (59).

In relation to vascular reactivity, a study conducted in 10 GHD-treated adolescents, 12 GHD untreated adolescents and 14 controls, noted a lower flow-mediated endothelium-dependent increase in the diameter of the brachial artery during hyperemia in untreated subjects. In addition, the hyperemia-induced blood flow increase was higher in treated patients than in controls and in untreated GHD adolescents. The presence of such vascular abnormalities, together with the increased epicardial adipose tissue thickness, lead to an increased cardiovascular risk in non-treated subjects, even though it is reversible after rh-GH (60). Abnormal vascular reactivity in young GHD adults was also confirmed in a previous study, which demonstrated a reduction of the brachial artery vasodilation induced by specific vasodilators (acetylcholine and sodium nitroprusside) in seven childhood-onset GHD patients. However, these vascular abnormalities were not confirmed in subjects with CO-GHD who had received adequate rh-GH, confirming that GHD was a trigger (61).

Finally, rh-GH can also influence blood pressure. Most of the studies argue that rh-GH induces a reduction in diastolic blood pressure and no change in systolic blood pressure, through an increase in nitric oxide formation, stimulation of the renin-aldosterone system and decrease in intima-media thickness (62). To date, there is only a study in which rh-GH was clearly associated with hypertension (63).

### GH and Gonadal Function

The effect of rh-GH on gonadal function has been widely investigated, since it influences the hypothalamic-pituitary-gonadal axis, facilitating the release of GnRH and consequently of gonadotropins (64).

In 1994 our group described the effect of the rh-GH on the improvement of spermatogenesis in 10 infertile patients presenting with idiopathic severe oligozoospermia, normogonadotropinemia, or moderate hypergonadotropinemia, with low IGF-1 values or values within the lower limit of the normal range. Short-term rh-GH led to an improvement in both sperm concentration and motility in 50% of subjects (65). In a subsequent paper, we ruled out the possibility of prolonged rh-GH use negatively affecting testicular development or function (66).

In women, IGF-1 plays a role in the proliferation and differentiation of granulosa cells and stimulates steroidogenesis in large follicles and theca cells. Furthermore, a recent study showed that GH, along with IGF-1, interacts with local ovarian factors, such as VEGF-A and FGF-2, thus being a necessary actor in the ovarian angiogenesis (67).

## Impact on Quality of Life

The impact of rh-GH on the quality of life (QoL) of GHD patients during the transition period seems less clear than in adulthood.

However, Abs and co-authors showed a positive relationship between stature gain after rh-GH in childhood and the improved QoL during the transition, while a negative relationship between the duration of rh-GH discontinuation and QoL was reported (34). Another paper evaluated through a survey the impact of rh-GH on QoL at baseline and after 1 and 2 years of rh-GH, finding that body shape and sexual arousal were significantly lower after rh-GH suspension, thus negatively affecting the QoL (68). In contrast to Mauras’ paper (26), several studies agreed that rh-GH is effective in improving QoL, with a significant positive change in health-related aspects (68, 69).

Finally, the effect of therapy resumption was studied in another couple of studies, which showed a worsening of the QoL after 1 year of rh-GH suspension, counterbalanced following 6 months of rh-GH resumption (70, 71).

## What Is the Right Therapeutic Dosage During the Transition?

During the transition, the tendency is to start with an intermediate dose between 0.22 and 0.30 mg/kg/week as in childhood and 0.01–0.1 mg/kg/week as in adulthood (72).

In case the therapy was suspended after reaching final height, it should be resumed at a dosage of 0.21 mg/kg/week, and then titrate based on age, IGF-1 values, clinical response and the possible appearance of adverse effects (73). However, based on our clinical practice, we recommend starting with the lowest therapeutic dose and then adjust dosage according to IGF-1 levels, clinical response and absence (even minimal) of adverse effects. **Table 2** summarises the possible main side effects associated with rh-GH.

**Table 2:** Main side effects associated with rh-GH.

Side effects	
Headache
Glucose disorders
Insulin resistance
Idiopathic intracranial hypertension
Increased intraocular pressure
Arthralgia

In women receiving oral oestrogen replacement therapy, higher doses of rh-GH are typically required, as oral oestrogens seem to attenuate the metabolic actions of GH on its liver receptor, lowering IGF-1 secretion. It would therefore be preferable to use transdermal oestrogens, in order to avoid/attenuate the effect on the liver (74).

Regarding the production of thyroid hormones, rh-GH can cause a slight reduction in FT4 and TSH levels, and an increase in FT3 and could therefore unmask central hypothyroidism (75). Therefore, before starting rh-GH and during treatment, thyroid function should be monitored closely, particularly in the first 6 months.

At the adrenal level, GH can reduce the activity of the enzyme 11βHSD1, resulting in reduced conversion of cortisone to cortisol. Thus, an assessment of the HPA axis function (through the evaluation of basal AM and PM cortisol levels and, when needed, after stimulation, e.g., ACTH test or ITT) should be made before and after starting rh-GH, as GHD could mask the presence of a hidden central hypoadrenalism (76).

Rh-GH, even during the transition, must be carefully monitored to avoid the onset of possible adverse events. Every 6 months, it is advisable to carry out a haematochemical screening, with the evaluation of IGF-1, serum glucose, HbA1c and lipid profile, and a clinical evaluation based essentially on the measurement of weight and waist circumference and blood pressure. Every year a possible improvement in bone mineral density through a DXA scan, and of the intima media thickness by ultrasound examination should be evaluated, as well as any positive changes in QoL (12).

## GH Treatment in Cancer Survivors During Transition

GHD is the most frequent endocrine disorder in childhood cancer survivors, especially in those with a history of pathology (and treatments) of the hypothalamic-pituitary region (20). In this respect, there is no unequivocal data on the risk of recurrence, as several studies indicate a very low or no-risk (77–80), whereas others provide no certain conclusions (81). The same risk of recurrence in GH-treated cancer survivors compared to non-treated subjects was found in two different studies (82, 83).

Others found no increased risk of cancer recurrence in a rh-GH treated paediatric population (84) and in patients with previous brain cancer (85, 86). As to the risk of developing a secondary neoplasm, a recent meta-analysis indicated that rh-GH seems not increase this risk (87). However, opposite results were found in other relevant studies, such as CCSS, GeNeSIS, and HypoCCS, in which the percentage of second neoplasms was 3.8% in GeNeSIS and 6% in HypoCCS (77, 88).

The Endocrine Society suggests starting rh-GH 1 year after stopping cancer therapy, when there is no more evidence of cancer disease. In the case of chronic or not totally eradicable oncological diseases, the choice whether to start rh-GH or not should be tailored according to the characteristics of the tumor and the patient, after a proper discussion with the oncologist (20).

A very controversial topic is the choice of the therapeutic rh-GH dose. However, it should be appropriate to use the minimum effective dose so as to decrease symptomatology. The most widely used and free of side effects therapy is the daily administration of 0.21 mg/kg/week, adjusted so as to reach normal IGF-1 levels (89).

To summarise, papers published so far show that somatotropin is indicated in those patients who have GHD and it appears safe in terms of tumor recurrence. Of course, caution is extremely important in the follow-up of GHD patients during the transition period, but it would be inappropriate to deprive them of therapy that is central to treating this deficiency.

## Conclusions

Transition is a period in life in which the maturation of the organism is completed. During the transition, GH plays a relevant role on bone maturation, metabolism and body composition. GHD increases cardiovascular risk and impacts fertility negatively. For these reasons, GH is considered extremely important for a good quality of life. There are still uncertainties about when and how to re-test to confirm or not a persistent GHD in patients previously treated with somatotropin, and there is still no total agreement regarding the therapeutic dosage. The link between rh-GH and tumor recurrence in cancer survivors is not totally clear. However, most of the studies report no evidence of increased recurrence risk in these patients during the transition.

Further studies with a longer duration of rh-GH are needed, in order to assess with more accuracy, the effects of rh-GH during the transition, and eliminate those uncertainties that are still present on this fascinating but often too feared hormone.

## Author Contributions

All the authors have made a substantial, direct and intellectual contribution to the work. MS revised it critically. All authors approved the work for publication. All authors contributed to the article and approved the submitted version.

## Conflict of Interest

The authors declare that the research was conducted in the absence of any commercial or financial relationships that could be construed as a potential conflict of interest.
